# Burnout of emergency nurses in a South African context: the role of job demands and resources, and capabilities

**DOI:** 10.3389/fpsyg.2023.1119063

**Published:** 2023-05-18

**Authors:** Neil B. Barnard, Sebastiaan Rothmann, Leon T. De Beer, Welma Lubbe

**Affiliations:** ^1^Optentia Research Unit, North-West University, Vanderbijlpark, South Africa; ^2^WorkWell Research Unit, North-West University, Potchefstroom, South Africa; ^3^Department of Psychology, Norwegian University of Science and Technology, Trondheim, Norway; ^4^Quality in Nursing and Midwifery Research Focus Area, North-West University, Potchefstroom, South Africa

**Keywords:** sustainable employability, job demands, job resources, work capabilities, burnout, emergency nurse, person-centered, South Africa

## Abstract

Emergency nurses are prone to burnout due to the nature of their profession and working environment, potentially putting their sustainable employability at risk and so too the care provided by and success of emergency departments. Psychological research has predominantly focused on samples drawn from western, educated, industrialized, rich, and democratic (WEIRD) societies, concerning a small part of the world population. Consequently, this study investigated emergency nurses' burnout in a non-WEIRD society and assessed the role of job demands-resources and work capabilities on their burnout levels. A total of 204 emergency nurses in a South African context participated in a cross-sectional survey. The Job Demands-Resources Scale, the Capability Set for Work Questionnaire, and the Burnout Assessment Tool—Short Form were administered. Using and developing knowledge and skills and building and maintaining meaningful relationships were the strongest work capabilities of emergency nurses. In contrast, earning a good income, involvement in important decisions, and contributing to something valuable were the weakest capabilities. Latent class analysis resulted in three capability sets: a robust capability set, an inadequate capability set, and a weak capability set. Regarding job resources, emergency nurses with a robust capability set reported better relationships with their supervisors and higher job autonomy than the inadequate and weak capability sets. In addition, emergency nurses with a robust capability set reported better co-worker relationships and better access to good equipment than those with a weak capability set. Nurses with an inadequate capability set experienced significantly more challenging job demands than the other two sets. Finally, nurses with a weak capability set (compared to the robust capability set) experienced significantly higher levels of exhaustion and mental distance. Improving emergency nurses' job resources (especially relationships with co-workers and supervisors, job autonomy, and equipment sufficiency) would increase their capabilities, decreasing their burnout levels, especially exhaustion and mental distance.

## 1. Introduction

The work environment is one of the most significant stressors for emergency nurses^1^ (McDermid et al., 2020). In a fast-paced (Abellanoza et al., 2018), ever-changing, dynamic, and highly regulated environment, they must initiate treatment for a broad spectrum of illnesses, some of which are life-threatening. Emergency nurses must navigate recurring critical incidents (such as severe injuries, death, and resuscitation), staffing shortages, overcrowding, unpredictable (McDermid et al., 2020) and high workloads (Kato et al., 2021), and physical demands (Castner, 2019), often without sufficient job resources. In addition, they must deal with emotionally charged distressed family members, friends, or peers of patients (Hogan et al., 2016), safeguard psychiatric patients waiting to receive the appropriate care (Castner, 2019), and deal with human trafficking victims coming through the emergency department of the hospital (Dols et al., 2019). Moreover, the COVID-19 pandemic has introduced new challenges for emergency nurses, such as the fear of infecting people, organizational concerns (such as continuous reallocations and ever-changing protocols, new personal protective equipment, and quarantined professionals), support for novice nurses, and increased conflict (García-Martín et al., 2021).

The above-mentioned job demands, associated with a lack of job resources, can lead to an increased likelihood of burnout in emergency nurses (McDermid et al., 2020; Lluch et al., 2022), especially if they lack work capabilities. While socio-demographics associated with lifestyle, and personality (type A personality and need for social support) are contributing factors, work-related factors appear to be the most significant antecedents to burnout (Manzano-García and Ayala, 2017). Lack of recognition and promotion, high workload, low remuneration, conflict, and role ambiguity are some of the most prevalent work-related antecedents (see Manzano-García and Ayala-Calvo, 2014). However, further studies on other possible factors explaining burnout, such as lack of recognition, feminine stereotype, or excessive bureaucracy (Manzano-García and Ayala, 2017), and work capabilities (Gloss et al., 2017) are needed.

Burnout has been identified as one of the most significant risks facing the nursing profession, reaching alarming heights, with significant negative personal and organizational outcomes (Manzano-García and Ayala, 2017). Nurses suffer from increased emotional exhaustion and depersonalization, coupled with low personal fulfillment (Manzano-García and Ayala-Calvo, 2014). Research regarding the variables associated with the burnout of emergency nurses is essential because burnout negatively affects the quality of care they provide and their productivity. Also, burnout leads to increased turnover and decreased mental and physical health (Abellanoza et al., 2018), which may put the sustainable employability of emergency nurses at risk, producing negative organizational implications such as financial losses and a decreased ability to provide proper care to the society (Manzano-García and Ayala-Calvo, 2014).

Psychological research has predominantly focused on marginal samples comprising western, educated, industrialized, rich, and democratic (WEIRD) societies, arguably non-representative of the larger world population (Muthukrishna et al., 2020). Furthermore, studies regarding the burnout of emergency nurses have predominantly focused on high workload, poor co-worker and supervisor relationships and support, inefficient communication and teamwork, and staffing issues, utilizing models such as the job demands-control model (Karasek, 1979; Guthier et al., 2020) and the job demands-resources (JD-R) model (Demerouti et al., 2001; Bakker and de Vries, 2021). Values and contextual factors that impact an individual's ability to work are inadequately considered in these models (van der Klink, 2019). Rather than being seen as something meaningful with personal and social value that enables people to flourish, work is seen as a production factor in most economic and social theories (van der Klink, 2019). However, given the choice, people will more often than not seek work aligned with their preferences and character, and which feels good to them.

The study addresses the following gaps in the literature: First, it provides scientific information regarding the associations between emergency nurses' job demands and resources and work capabilities. The JD-R model provides a framework for investigating the work functioning of emergency nurses, but does not consider the effects of job demands and resources (i.e., means) on work capabilities (i.e., opportunities). The capability approach (CA; Sen, 1985) provides a framework for conceptualizing work capabilities. Building on the CA, van der Klink et al. (2016) pointed out that individuals can achieve tangible opportunities (in the form of capabilities) throughout their working lives. However, research information is needed regarding the links between job demands and resources and the work capabilities of emergency nurses. Second, no scientific information is available regarding the associations between job demands and resources, work capabilities, and burnout of emergency nurses. The sustainable employability (SE) model (van der Klink et al., 2016) provides a framework linking job demands-resources, work capabilities, and functioning using the CA (Sen, 1985; Abma et al., 2016). Sustainable employability allows them to “make a valuable contribution through their work, now and in the future, while safeguarding their health and welfare” (van der Klink et al., 2016, p. 74).

## 2. The capability approach

The capability approach (CA; Sen, 1985) offers an ethical framework that considers social justice and suggests the freedoms people enjoy in choosing the life they have reason to value, not purely on available resources (Abma et al., 2016; Sferrazzo and Ruffini, 2021). The CA consists of three components: functionings, capabilities, and agency (or freedom). Capabilities refer to a person's realized functionings [i.e., beings (or states) and doings (or activities)] within a given context. Agency refers to a person's opportunity to shape and achieve a life that that individual has reason to value. Thus, a capability is defined as a set of realized beings and doings a person had the freedom to choose by drawing on widespread opportunities to ultimately achieve valued outcomes, while considering personal characteristics and external factors (Robeyns, 2017).

### 2.1. Capabilities

People are increasingly looking for work that is valuable to them (i.e., valued work). The CA offers a framework in which emergency nurses' work values can be identified and, importantly, in which it can be determined whether they are enabled and able to achieve them (van der Klink et al., 2016). Recognizing their work as valuable (i.e., meaningful work) contributes significantly to the wellbeing and sustainable employability of emergency nurses (Van Casteren et al., 2021). Work values become capabilities when employees consider them important, are enabled to achieve them, and achieve them (van der Klink, 2019). Emergency nurses need to experience personal, social, and environmental conditions in which they are offered the freedom and opportunity to continually make meaningful contributions to the world, without endangering their health and wellbeing (Abma et al., 2016). Freedoms and opportunities to realize work outcomes that emergency nurses have reason to value mark the CA as a framework for studying employee wellbeing and the conditions required for sustainable employability. A fundamental premise on which the SE framework was built is that people need to possess a variety of opportunities to realize functionings they have reason to value (Dalziel et al., 2018).

A capability set is an aggregation of available options that reflect several states and activities people value (Sen, 1992). Sen (2008) has argued that a person's capability set should not be compared to a predetermined list. Instead, a proceduralist approach must be followed, focusing on objective and not subjective evaluations. However, while the CA can objectively reflect important social and material conditions and affordances, it lacks in portraying people's lives based on subjective values. Accordingly, Nussbaum (2011) has argued that a list is required to act as a guiding principle regarding individual values. Sferrazzo and Ruffini (2021) also suggested a list of work capabilities, but without any empirical evidence. Subsequently, Nussbaum (2011) has created a list of capabilities that are arguably vital for a person to flourish. Considering the above arguments by Sen and Nussbaum, this study followed a combined approach by incorporating Sen's (1985) and Nussbaum's (2011) approach to investigate the capability set of emergency nurses.

Within the SE framework (van der Klink et al., 2016), seven work capabilities are identified: using knowledge and skills; developing knowledge and skills; involvement in important decisions; building and maintaining meaningful contacts at work; setting own goals; earning a good income; and contributing to something valuable. When an emergency nurse considers a capability aspect (a) important, (b) enabled, and (c) achieved, that capability aspect is deemed part of the nurse's capability set (Abma et al., 2016). While a single capability is important, the true value is within a combined set (van der Klink, 2019). However, in the SE framework by van der Klink et al. (2016), the capability set does not solely concern an employee's abilities, but also the workplace opportunities available to the employee in achieving valued work outcomes. A person-centered approach allows for investigating whether different subgroups of persons experience similar capabilities. Because of this, person-centered techniques can be used to investigate capability sets among emergency nurses (Collie et al., 2020).

A study investigating the sustainable employability of teachers with hearing loss revealed that the sampled teachers considered using knowledge and skills as the most important value, regardless of hearing ability. However, the lowered score among teachers with hearing loss on achieving this work value indicated that they more frequently encountered challenges in using their knowledge and skills at work (Schriemer et al., 2022). Among South African secondary school teachers, using knowledge and skills, building and maintaining meaningful relationships at work, and contributing to something valuable were the most prevalent capabilities. Earning a good income, involvement in important decisions, and developing knowledge and skills were the least prevalent (De Wet and Rothmann, 2022).

Abma et al. (2016) and De Wet and Rothmann (2022) found that a capability set (rather than individual work capabilities) is a stronger predictor of wellbeing and performance. Investigating the different capability sets among emergency nurses and their association with their job demands and resources, as well as functioning (such as burnout), can provide valuable information on their wellbeing and conditions for sustainable employability. Therefore, this study investigated the associations of job demands and resources, capability sets, and burnout of emergency nurses.

### 2.2. Job demands and resources

A significant advancement in occupational health has been that of the job-demands resources (JD-R) model (Demerouti et al., 2001). It explains how burnout results from unfavorable working conditions (excessive job demands and insufficient job resources), leading to undesired outcomes (Lesener et al., 2019). The JD-R model is comprehensive, with a broad, flexible scope that considers risks and opportunities and includes all relevant job characteristics, acting as a helpful communication tool with stakeholders, with an accent on, and elaboration of resources (van der Klink, 2019). However, valued work, which is positively related to continued work, is not one of the focus points of the JD-R model. Valued work is personified by a balance between what people can do and be at work that they have reason to value (van der Klink, 2019).

According to the JD-R model, employment has job demands (the “bad” aspects that drain an employee's energy) and job resources (the “good” aspects). The model argues a health impairment process in which excessive job demands, coupled with insufficient job resources, lead to burnout, producing adverse outcomes, and a motivational process in which sufficient job resources lead to work engagement with positive outcomes (Schaufeli, 2017). Job demands may potentially not be perceived the same among emergency nurses. Some job demands may be regarded as hindrances, whereas others present as challenges (Bakker and Sanz-Vergel, 2013). Although job demands lead to burnout (Vinod Nair et al., 2020; Sawhney and Michel, 2022), how persons appraise job demands (as a hindrance or demand) moderates the extent of this (Li et al., 2020).

The relationship with, and support of, supervisors affect emergency nurses' wellbeing (Babamiri et al., 2021), engagement (Kato et al., 2021; De Wijn et al., 2022), and intentions to leave (Mirzaei et al., 2021). Combining co-worker and supervisor support and job autonomy can improve work-life balance, producing higher job satisfaction among emergency nurses (Rashmi and Kataria, 2021). Furthermore, the availability of, and ease of access to, appropriate equipment (Osborne et al., 2021), with a proper design and low failure rate, assist emergency nurses in providing safe patient care (Hu et al., 2021).

Studies investigating the relationships of job demands and resources with capabilities showed positive associations with task characteristics, relationship with supervisor, remuneration, performance support (Murangi et al., 2022), person-job fit, strengths use, and opportunity to craft (Gürbüz et al., 2022). Consequently, emergency nurses' job demands and resources were expected to be associated with capabilities.

Depending on employees' values, job demands and resources may have adverse or beneficial effects on capabilities (van der Klink, 2019). For example, a job demand (such as maintaining records) may be engaging if an individual has strong administrative skills, and values knowledge and skills. However, this demand may burden an individual who values meaningful relationships with others. Job resources are valuable because they affect the being and doings of individuals when they use or convert them (Sen, 2009). Through conversion factors, employees can convert job resources into capabilities (Robeyns, 2017). For instance, job autonomy may contribute to converting job demands into values (e.g., developing new knowledge and skills or contributing something valuable to society). In addition, job resources may reflect capabilities such as involvement in important decisions and setting own goals (van der Klink, 2019) or contribute to capabilities by providing opportunities for enablement and achievement. However, while job resources are necessary to function well, they are insufficient, unless an individual can convert them into capabilities.

### 2.3. Burnout as a functioning

The COVID-19 pandemic rejuvenated the focus on employee wellbeing, producing increased interest in burnout, which, as of 2022, has formed part of the International Classification of Diseases (ICD-11) (De Beer et al., 2022). Burnout has been classified as an occupational phenomenon referring to a “work-related state of exhaustion that occurs amongst employees, which is characterized by extreme tiredness, reduced ability to regulate cognitive and emotional processes and mental distancing” (Schaufeli et al., 2020, p. 4). Accordingly, burnout consists of four components: exhaustion, mental distance, emotional impairment, and cognitive impairment. Exhaustion implies an inability to exert effort (feeling depleted), while mental distance implies a lack of interest. Cognitive and emotional impairment refers to a reduced ability to regulate cognitions and emotions, respectively (Schaufeli et al., 2020).

A vast amount of research on burnout has confirmed its associated adverse effects on healthcare providers, with high burnout levels being more prominent among physicians and nurses due to high workload, long journeys, and ineffective interpersonal relationships (Garcia et al., 2019). Subsequently, burnout is a prevalent concern among emergency nursing professionals (Phillips et al., 2022). An unpredictable and high-stress work environment (Sexton et al., 2021), characterized by traumatic events, and unfavorable job characteristics such as varying work schedules, work and time pressures, low job control, and lack of social support are some of the most significant antecedents of burnout among these professionals (Adriaenssens et al., 2015). In recent times, the additional stressors of the COVID-19 pandemic have resulted in an increased risk of burnout among emergency nurses (Alanazi et al., 2021; Gualano et al., 2021; Phillips et al., 2022). Changes in workload (Butera et al., 2021), hospital resource shortages, worry regarding COVID-19, and stigma (Gualano et al., 2021) are some pandemic-related variables associated with increased burnout. Furthermore, support from co-workers and supervisors reduces the risk of burnout in emergency nursing personnel (Butera et al., 2021).

Burnout holds adverse outcomes for not only emergency nurses, but also the hospital and society due to poorer patient safety (Salyers et al., 2017; Garcia et al., 2019), perceived healthcare quality (including patient satisfaction), and quality indicators (Salyers et al., 2017). Emotional exhaustion has the most significant impact on quality and may be the most critical burnout constituent for intervention (Salyers et al., 2017). Interventions aimed at creating organized workflows, leading to increased autonomy at work for healthcare professionals, can foster better patient safety practices (Garcia et al., 2019). Addressing burnout among emergency nurses needs to be a priority for hospital and emergency nursing management (Abellanoza et al., 2018), especially following a viral outbreak resulting in a global pandemic.

Within the framework of SE, an insufficient capability set can result in emergency nurses' burnout. The capability set for work has been found to positively affect work engagement (Gürbüz et al., 2022), an antipode of burnout (Schaufeli et al., 2008; Wickramasinghe et al., 2021). Subsequently, emergency nurses' capabilities can potentially impact their burnout.

## 3. Current study

The SE model argues that emergency nurses not only require sufficient job resources, but also need to convert these to achieve valued work outcomes. Subsequently, job resources hold value for people regarding what they can be or achieve when they use or convert said resources (Sen, 2009). However, based on the CA and the SE model it is argued that burnout is not a matter of the possession of resources, but rather of what an individual is able to do and to be (see Wolff and De-Shalit, 2007). Emergency nurses not only need resources to remain sustainably employed, but also need to be able to take advantage of them. They can do so by converting resources into opportunities via conversion factors, which are essential to achieving valued work functionings. Conversion factors have the potential to illuminate aspects that have an impact on the capabilities and functionings of emergency nurses. For instance, autonomy may not be as efficient if nurses are reluctant to take control of their duties or shape their jobs. Conversion factors can also indicate why emergency nurses do not function optimally, regardless of having access to relevant, abundant resources (Robeyns, 2017; van der Klink, 2019).

**Figure 1:**
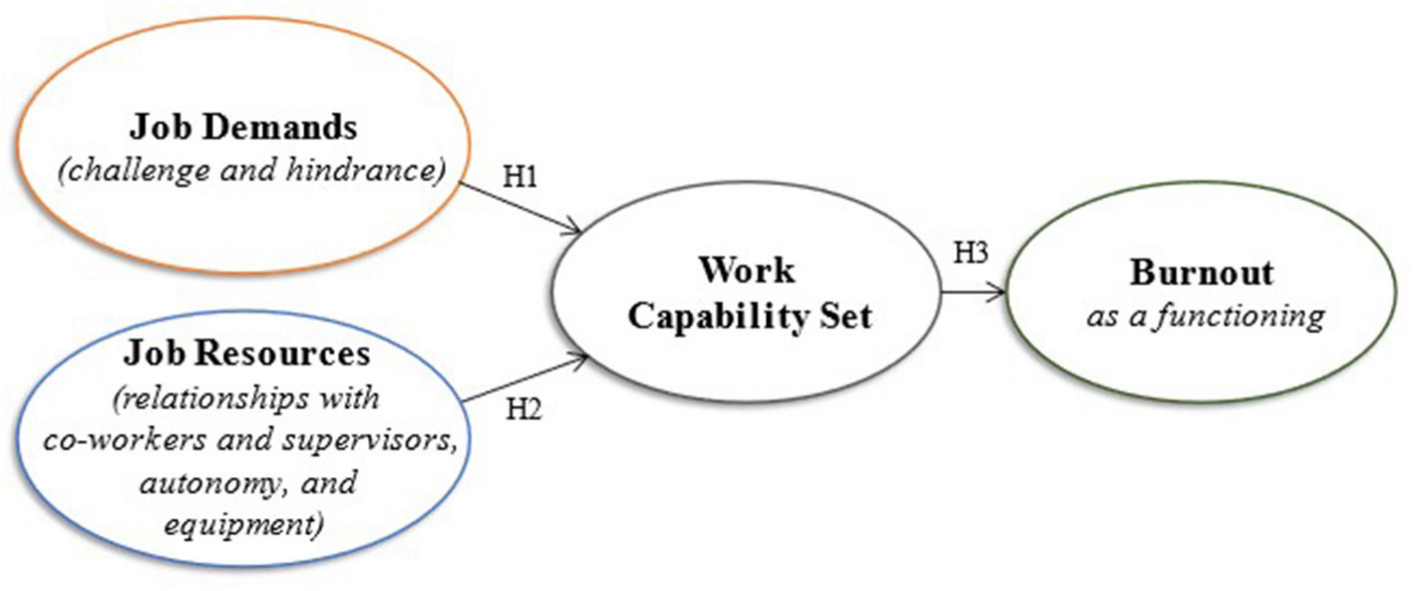
Model of job demands and resources, capabilities, and burnout.

Therefore, this study also investigated the differences in associations among emergency nurses' job demands and resources, capabilities, and burnout in different biographical groups. While there is ample empirical support for the use of the JD-R model in investigating employee wellbeing (Schaufeli, 2017; Lesener et al., 2019), there is a need to understand the relationship of emergency nurses' work capabilities with their job demands and job resources, and the association thereof on their capabilities and functioning. For this reason, the study investigated emergency nurses' sustainable employment by incorporating their job demands, job resources, capabilities, and burnout within the SE framework, as illustrated in Figure 1.

The following hypotheses were set:

Hypothesis 1 (H1): Job demands of emergency nurses are negatively associated with their work capabilities.
Hypothesis 2 (H2): Job resources of emergency nurses are positively associated with their work capabilities.
Hypothesis 3 (H3): Emergency nurses' burnout is negatively associated with their work capabilities.

## 4. Methods

### 4.1. Research design

A quantitative research approach with a cross-sectional survey design was implemented. As the concept of work capabilities is a reasonably novel phenomenon, especially among South African emergency nurses and as far as its association with job demands, job resources, and burnout is concerned, a cross-sectional survey may serve as a good basis for theory and the target for intervention (Spector, 2019).

### 4.2. Participants

The South African healthcare system comprises two parallel systems: a public healthcare system and a private one. The public sector is government-funded, and most of the population depends on the public sector for care (around 71%). The private sector is predominantly funded through medical aid schemes and health insurance paid for by the individual (Rensburg, 2021). Nurses working in the emergency department of hospitals providing Level 1 or 2 trauma care within the Gauteng province of South Africa were invited to participate in the study. A Level 1 trauma care hospital can provide leadership and total care for every aspect of injury (prevention to rehabilitation) while having 24-h availability of all primary specialties and a trauma surgeon as director. While a Level 2 trauma care hospital can provide initial definitive trauma care irrespective of injury severity (including the typical specialties), with a 24-h medical cover (Trauma Society of South Africa, 2021). Thirteen hospitals, consisting of four private hospital groups and one public hospital, were included in the study. A total of 204 emergency nurses provided satisfactory responses to the survey. The characteristics of the participants are reported in Table 1.

**Table 1:** Characteristics of participants (*N* = 204).

**Demographic variable**	**Grouping**	***n***	**%**
Gender	Male	54	26.47
	Female	146	71.57
	Missing values	4	1.96
Age	18–29 years of age	27	13.24
	30–39 years of age	58	28.43
	40–49 years of age	30	14.71
	50–59 years of age	18	8.82
	60+ years of age	2	0.98
	Missing values	69	33.82
Highest qualification	Grade 12 (NQF 4)	19	9.31
	Higher certificate (NQF 5)	53	25.98
	Three-year diploma (NQF 6)	49	24.02
	Bachelor's degree (NQF 7)	42	20.59
	Honors degree (NQF 8)	28	13.73
	Master's degree (NQF 9)	1	0.49
	Other	5	2.45
	Missing values	7	3.43
Contract type	Permanent contract	153	75.00
	Fixed-term contract	17	8.33
	Agency placement	30	14.71
	Missing values	4	1.96

Table 1 shows that the participants were mostly female (71.57%), aged between 30 and 49 years (43.14%). Most of the participants' highest level of education was a higher certificate (25.98%), 3-year diploma (24.02%), or bachelor's degree (20.59%). They were predominantly permanently employed (75%).

### 4.3. Measuring instruments

The *Job Demands-Resources Scale* (JDRS; Rothmann et al., 2006) was administered to measure emergency nurses' job demands and resources. The study included 20 items: eight to measure job demands and 12 to measure job resources. Job demands included two subscales: *challenge* (four items, e.g., “My job requires me to work very hard.”) and *hindrance* (four items, e.g., “I have to go through a lot of red tape to get my job done”). Job resources included four subscales: *relationship with co-workers* (three items, e.g., “Can you count on your co-workers when you come across difficulties in your work?”), *relationship with supervisor* (three items, e.g., “Do you get on well with your supervisor?”), *autonomy* (three items, e.g., “Does your job offer you the possibility of independent thought and action?”), and *equipment* (three items, e.g., “Do you have sufficient equipment to do your work tasks?”). The items were rated on a Likert scale, ranging from 1 (*never*) to 5 (*always*). Research showed that the scale was valid, reliable, and equivalent across different organizations (Rothmann et al., 2006). More recently, it was found that the scale had retained internal consistency (ranging from 0.76 to 0.92) in a South African context (Janse van Rensburg and Rothmann, 2020).

The *Capability Set for Work Questionnaire* (CSWQ; Abma et al., 2016) was used to measure emergency nurses' work capabilities. The CSWQ measures seven work capabilities: (1) using knowledge and skills, (2) developing knowledge and skills, (3) involvement in important decisions, (4) meaningful relationships at work, (5) setting own goals, (6) earning a good income, and (7) contributing to something valuable. For each of these seven capabilities, the emergency nurses were requested to indicate whether (a) they considered the work value important (*importance*; e.g., “How important is it to you to be able to use your knowledge and skills at work?”), (b) their work was offering them sufficient opportunities to achieve it (*enablement*; e.g., “Does your current work offer you enough opportunities to do that?”), and (c) they succeeded in achieving it (*achievement*; e.g., “To what extent do you succeed in doing so?”). The items were rated on a Likert scale, ranging from 1 (*totally not*) to 5 (*to a very great extent*). The CSWQ has convergent, predictive, and incremental validity (Gürbüz et al., 2022) and is reliable (ω = 0.77) (Murangi et al., 2022).

The *Burnout Assessment Tool*—Short Form (BAT-12; Schaufeli et al., 2020) was utilized to measure emergency nurses' levels of burnout. The BAT-12 consists of 12 items measuring four components of burnout, with three items each. The four components are *exhaustion* (e.g., “At work, I feel mentally exhausted.”), *mental distance* (e.g., “I struggle to find any enthusiasm for my work.”), *cognitive impairment* (e.g., “At work, I have trouble staying focussed.”), and *emotional impairment* (e.g., “At work, I feel unable to control my emotions.”). The items were rated on a Likert point scale, ranging from 1 (*never*) to 5 (*always*). In a study investigating the psychometric properties of the scale among the South African working population, De Beer et al. (2022) found the BAT-12 to be reliable, valid, and invariant across gender and ethnicity. The scale illustrated convergent validity and proved a robust instrument with good psychometric properties to measure burnout. The factor loadings ranged from 0.47 to 0.96, and the McDonald's omega reliability scores were acceptable (exhaustion = 0.88, mental distance = 0.71, cognitive impairment = 0.86, and emotional impairment = 0.87).

### 4.4. Research procedure

The researchers obtained scientific clearance for the study from the North-West University Industrial Psychology and Human Resource Management (IPSHRM) Scientific Committee on 14 July 2021 and ethics clearance from the North-West University Health Research Ethics Committee (NWU-HREC) on 5 February 2022 (NWU-00273-21-A1). After that, permission was obtained from four private hospital groups and the corresponding hospital and emergency department management. Regarding the participating public hospital, permission was obtained from the Provincial Department of Health. The researcher collected data using an online platform (i.e., QuestionPro) and by distributing hard-copy booklets at the participating hospitals. The research purpose was explained. Furthermore, the researchers explained that participation in the study was voluntary and that all information and responses would be kept confidential and anonymous. Participation was predominately through hard-copy booklets (91.18%).

### 4.5. Data analysis

Data analysis was done using SPSS Version 27 (IBM Corporation, 2020) and Mplus Version 8.8 (Muthén and Muthén, 1998–2017). Various goodness-of-fit indices and information criteria to assess model fit were employed to ultimately select the model that fitted the data best (West et al., 2012): the chi-square statistic (the test of absolute fit of the model), standardized root mean residual (SRMR), root mean square error of approximation (RMSEA), Tucker-Lewis index (TLI), and comparative fit index (CFI). TLI and CFI values higher than 0.90 indicate an acceptable value, with a value higher than 0.95 indicating an excellent fit. For SRMR and RMSEA values to be acceptable, a score below 0.08 is required with a 90% confidence interval, not including absolute zero (Wang and Wang, 2020).

Scale reliability was investigated using McDonald's omega coefficient (ω). Pearson correlations were used to assess the associations between job demands and resources, the three capability constituents (i.e., importance, enablement, and achievement), burnout, and the relationships between job demands and resources and the seven work capabilities (Field, 2013). In addition, point biserial correlations were used to estimate the relationships of the three capability constituents (i.e., importance, enablement, and achievement) with the job demands-resources and burnout variables.

Latent class analysis (LCA) was implemented to determine different emergency nurse capability profiles through Mplus 8.8 (Muthén and Muthén, 1998–2017). The maximum likelihood with robust standard errors (MLR) was used to estimate multiple latent classes. A model was retained if it showed a significant improvement from the reference model to the model with more classes. Models were compared based on their Bayesian information criterion (BIC), Akaike information criterion (AIC), and sample-size adjusted Bayesian information criterion (aBIC) values (Kline, 2016; Wang and Wang, 2020). The decision to select the model with the optimal number of classes was made using the Lo-Mendell-Rubin test (LMR LR; Lo et al., 2001), the adjusted Lo-Mendell-Rubin test (aLMR), and the bootstrapped likelihood ratio test (BLRT; Wang and Wang, 2020). Entropy values (ranging from 0 to 1) were used to assess profile verification quality. An entropy value close to 1 indicates a suitable classification. Furthermore, the average latent class probabilities were investigated to determine the probability of correct class membership. A probability score above 0.80 is considered to be a good indicator of member probability (Geiser, 2013).

Then, Bolck-Croon-Hagenaars analysis (BCH; Bolck et al., 2004) was performed to compare different capability profiles with the job demands and resources. Multinomial regression analysis was used to determine demographical differences (i.e., gender, education, and contract type) between the identified latent capability profiles.

## 5. Results

### 5.1. Capabilities of emergency nurses

Frequency analysis was used to analyze emergency nurses' capabilities regarding importance, enablement, and achievement (see Table 2).

**Table 2:** Capabilities of emergency nurses.

**Capabilities**	**Mean**	**SD**	**Capabilities and components (percentage)**			
			**Capability**	**Importance**	**Enablement**	**Achievement**
UKS	0.68	0.47	67.65	94.12	78.43	76.96
DKS	0.63	0.48	62.75	91.67	68.63	69.61
IID	0.37	0.48	36.76	77.94	46.08	43.63
MRW	0.60	0.49	60.29	88.24	68.14	65.20
SOG	0.54	0.50	53.92	85.78	62.25	58.82
EGI	0.23	0.42	23.04	84.31	27.94	32.35
CSV	0.45	0.50	45.10	82.35	51.47	50.00

SD, standard deviation; UKS, using knowledge and skills; DKS, developing knowledge and skills; IID, involvement in important decisions; MRW, meaningful relationships at work; SOG, setting own goals; EGI, earning a good income; CSV, contributing to something valuable.

Table 2 shows that while most emergency nurses valued all seven work values (78–94%), some lacked enablement and achievement. The realized capabilities (i.e., valued, enabled, and achieved) that were most evident among the sample were using knowledge and skills (67.65%), developing knowledge and skills (62.75%), and meaningful relationships at work (60.29%), while earning a good income (23.04%) and involvement in important decisions (36.76%) were the least prevalent.

### 5.2. Measurement models of job demands, job resources, and burnout

The researcher used confirmatory factor analysis (CFA) to evaluate the measurement models of job demands (i.e., challenge and hindrance), job resources (co-worker relations, supervisor relations, autonomy, and equipment), and burnout (exhaustion, mental distance, cognitive impairment, and emotional impairment). The fit statistics obtained were as follows: χ^2^ = 671.01 (*df* = 418; *p* < 0.001), CFI = 0.96, TLI = 0.95, RMSEA = 0.05 (0.047, 0.062, *p* = 0.166), SRMR = 0.07. The sizes of the factor loadings of the items on their target factors were acceptable: challenge: λ = 0.60–0.87 (mean = 0.73), hindrance: λ = 0.61–0.96 (mean = 0.76), co-worker relations: λ = 0.75–0.91 (mean = 0.83), supervisor relations: λ = 0.86–0.94 (mean = 0.90), autonomy: λ = 0.68–0.86 (mean = 0.78), equipment: λ = 0.73–0.90 (mean = 0.81), exhaustion: λ = 0.72–0.83 (mean = 0.79), mental distance: λ = 0.20–0.91 (mean = 0.51), cognitive impairment: λ = 0.84–0.95 (mean = 0.89), and emotional impairment: λ = 0.78–0.89 (mean = 0.84). Therefore, the factors were well-defined and aligned with theoretical expectations.

### 5.3. Descriptive statistics, reliabilities, and correlations

The McDonald's omega reliabilities, means, standard deviations, and Pearson correlations of the variables used in the study are reported in Table 3. McDonald's omega coefficients above 0.70 were obtained for all the scales, indicating acceptable reliability (Nunnally and Bernstein, 1994).

**Table 3:** Descriptive statistics, reliabilities, and correlations of the scales.

**Variable**	**ω**	**Mean**	***SD***	**1**	**2**	**3**	**4**	**5**	**6**	**7**	**8**	**9**	**10**	**11**	**12**
1. Challenge demands	0.79	4.14	0.74	–	–	–	–	–	–	–	–	–	–	–	–
2. Hindrance demands	0.82	2.37	0.95	0.57^**^	–	–	–	–	–	–	–	–	–	–	–
3. Co-worker relationship	0.81	4.14	0.77	−0.12	−0.49^**^	–	–	–	–	–	–	–	–	–	–
4. Supervisor relationship	0.89	3.85	1.04	−0.19^**^	−0.39^**^	0.64^**^	–	–	–	–	–	–	–	–	–
5. Autonomy at work	0.78	3.60	0.90	−0.20^**^	−0.28^**^	0.61^**^	0.69^**^	–	–	–	–	–	–	–	–
6. Equipment	0.80	4.00	0.79	−0.14	−0.35^**^	0.50^**^	0.60^**^	0.68^**^	–	–	–	–	–	–	–
7. Importance	0.80	0.87	0.22	0.02	0.08	0.05	0.10	0.15^*^	0.10	–	–	–	–	–	–
8. Enablement	0.80	0.58	0.32	−0.12	−0.12	0.27^**^	0.42^**^	0.43^**^	0.31^**^	0.51^**^	–	–	–	–	–
9. Achievement	0.82	0.58	0.32	−0.11	−0.10	0.30^**^	0.38^**^	0.41^**^	0.29^**^	0.53^**^	0.81^**^	–	–	–	–
10. Exhaustion	0.79	3.16	0.95	0.45^**^	0.48^**^	−0.30^**^	−0.40^**^	−0.33^**^	−0.33^**^	−0.01	−0.21^**^	−0.18^*^	–	–	–
11. Mental distance	0.71	2.74	0.97	0.41^**^	0.55^**^	−0.41^**^	−0.49^**^	−0.40^**^	−0.41^**^	0.00	−0.20^**^	−0.19^**^	0.85^**^	–	–
12. Cognitive impairment	0.86	1.73	0.77	0.31^**^	0.54^**^	−0.40^**^	−0.36^**^	−0.27^**^	−0.37^**^	0.08	−0.02	−0.10	0.59^**^	0.81^**^	–
13. Emotional impairment	0.81	1.78	0.80	0.25^**^	0.50^**^	−0.40^**^	−0.34^**^	−0.29^**^	−0.37^**^	0.10	−0.03	−0.10	0.52^**^	0.73^**^	0.88^**^

SD, standard deviation; ^**^p < 0.01; ^*^p < 0.05; *r* < 0.30 = small effect; 0.30 < *r* < 0.50 = medium effect; *r* > 0.50 = large effect.

Table 3 shows that challenge demands were statistically significantly and positively related to three dimensions of burnout, namely, exhaustion, mental distance, and cognitive impairment (all medium effects). In addition, hindrance demands were statistically significantly related to four dimensions of burnout (all large effects). Furthermore, the four job resources (co-worker relations, supervisor relations, autonomy, and equipment) were negatively related to the four burnout dimensions (all medium effects).

As shown in Table 3, relationships with co-workers and supervisors, autonomy, and equipment were significantly and positively associated with the enablement and achievement components of capabilities (small to medium effects). The work value component was only significantly associated with autonomy (positive, small effect). Furthermore, enablement and achievement were negatively associated with exhaustion and mental distance (small effects).

Not shown in Table 3, autonomy at work and relationship with supervisors were associated with the most capabilities (*p* < 0.01; medium effects or higher). Autonomy was related to using knowledge and skills, developing new knowledge and skills, being involved in important decisions, building and maintaining meaningful work relationships, and contributing to something valuable. Relationship with supervisors was associated with being involved in important decisions, building and maintaining meaningful relationships at work, earning a good income, and contributing to something valuable. Challenge and hindrance demands were significantly negatively associated (*p* < 0.01) with building and maintaining meaningful relationships at work. Regarding burnout, there was only a significant negative relationship (*p* < 0.01) between exhaustion and mental distance, on the one hand, and earning a good income, on the other hand.

### 5.4. Latent class analysis

Latent capability classes were analyzed using binary outcomes after converting the participants' responses on the five-point Likert scale into binary codes: a response between 1 and 3 = 0 and between 4 and 5 = 1. Table 4 reports the results of the four capability profiles.

**Table 4:** Comparisons of different capability latent class analysis models.

**Classes**	**AIC**	**BIC**	**aBIC**	**Entropy**	**LMR LR test**	**aLMR LR test**	**BLRT**	**Smallest class**
					***p*-value**	***p*-value**	***p*-value**	**membership**
Class 1	1,866.28	1,888.50	1,866.33	–	–	–	–	–
Class 2	1,563.11	1,612.88	1,565.36	0.82	0.001^**^	0.001^**^	0.001^**^	48.53%
Class 3	1,528.39	1,604.71	1,531.83	0.79	0.003^**^	0.003^**^	0.001^**^	28.92%
Class 4	1,533.64	1,636.50	1,538.28	0.84	0.029^*^	0.031^*^	0.725	10.29%

AIC, Akaike information criterion; BIC, Bayesian information criterion; aBIC, adjusted Bayesian information criterion; LMR LR, Lo-Mendell-Rubin test; aLMR LR, adjusted Lo-Mendell-Rubin test; BLRT, bootstrapped likelihood ratio test; ^**^p < 0.01; ^*^p < 0.05.

As shown in Table 4, Class 3 reported superior fit indices compared to the other three classes. Class 2 fitted the data better than Class 1: ΔAIC = −303.17; ΔBIC = −275.62; ΔaBIC = −300.97, LMR LR (*p* < 0.01), aLMR (*p* < 0.01), BLRT (*p* < 0.01). Class 3 fitted the data better than Class 2: ΔAIC = −34.72; ΔBIC = −8.17; ΔaBIC = −33.53, LMR LR (*p* < 0.01), aLMR (*p* < 0.01), BLRT (*p* < 0.01). Class 4 reported inferior fit indices compared to Class 3: ΔAIC = 5.25; ΔBIC = 31.79; ΔaBIC = 6.45, LMR LR (*p* < 0.05), aLMR (*p* < 0.05), BLRT (*p* > 0.05).

**Figure 2:**
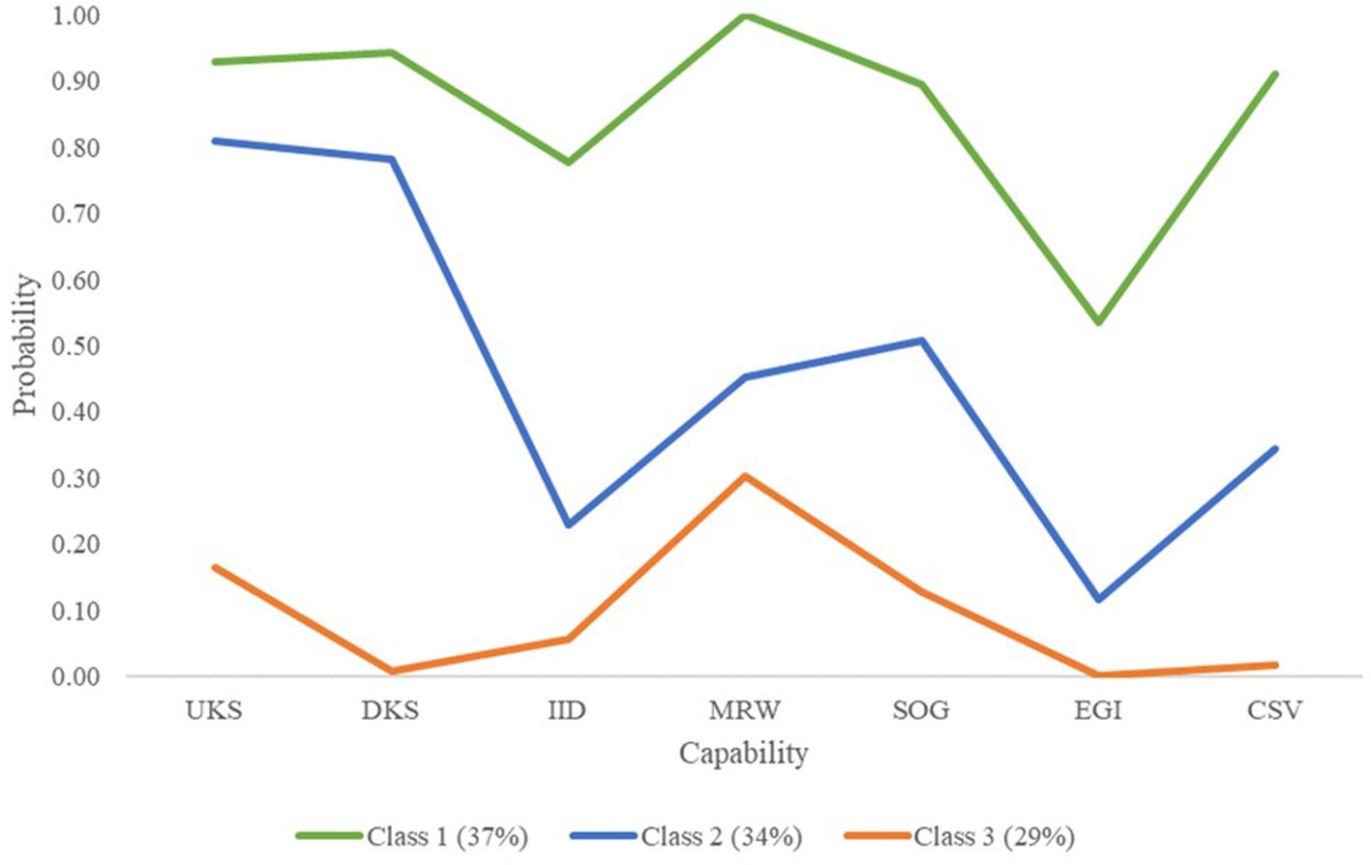
Latent profiles of capabilities of emergency nurses. UKS, using knowledge and skills; DKS, developing knowledge and skills; IID, involvement in important decisions; MRW, building and maintaining meaningful relationships at work; SOG, setting own goals; EGI, earning a good income; CSV, contributing to something valuable; Class 1, robust capability set; Class 2, inadequate capability set; Class 3, weak capability set.

Figure 2 illustrates the three identified emergency nurse capability sets. Class 1 had 76 (37.25%) members assigned to it, Class 2 had 69 (33.82%), and Class 3 had 59 (28.92%), indicating acceptable membership proportions. The average latent class probabilities were 0.88, 0.92, and 0.91 for Classes 1, 2, and 3, respectively. The entropy value was 0.79, indicating an acceptable classification (Wang and Wang, 2020). The bivariate standardized residuals were not statistically significant. Therefore, the assumption of local independence within classes (Sterba, 2013) was met (http://www.statmodel.com/discussion/messages/13/3895.html).

The classes depicted in Figure 2 can be interpreted as follows: (a) Class 1: robust capability set. Emergency nurses assigned to this class scored high on six capabilities and moderate on earning a good income. (b) Class 2: inadequate capability set. Emergency nurses in this group elicited high scores for using and developing knowledge and skills, moderate scores for meaningful relationships at work, setting own goals, and contributing to something valuable, and low scores for involvement in important decisions and earning a good income. (c) Class 3: weak capability set. Emergency nurses in this group had low scores for all capabilities.

Table 5 indicates the differences between the job demands and resources and four burnout components of the three capability sets.

**Table 5:** Comparisons of different capability profiles with burnout and job demands-resources.

**Variable**	**χ^2^**	***p***	**Class**	**Mean λ**	**SE**	**Class comparison**	**χ^2^**	***p***
Challenge demands	15.56	0.001^**^	1	−0.16	0.07	1 vs. 2	12.77	0.001^**^
			2	0.20	0.06	1 vs. 3	0.11	0.746
			3	−0.13	0.07	2 vs. 3	10.61	0.001^**^
Hindrance demands	1.42	0.492	1	−0.06	0.09	1 vs. 2	1.38	0.240
			2	0.08	0.08	1 vs. 3	0.23	0.629
			3	−0.01	0.08	2 vs. 3	0.63	0.427
Co-worker relations	13.39	0.001^**^	1	0.17	0.09	1 vs. 2	3.08	0.079
			2	−0.07	0.10	1 vs. 3	13.39	0.001^**^
			3	−0.25	0.08	2 vs. 3	1.83	0.176
Supervisor relations	36.70	0.001^**^	1	0.43	0.10	1 vs. 2	15.60	0.001^**^
			2	−0.21	0.11	1 vs. 3	33.73	0.001^**^
			3	−0.40	0.11	2 vs. 3	1.29	0.257
Autonomy	42.15	0.001^**^	1	0.31	0.07	1 vs. 2	9.39	0.002^**^
			2	−0.06	0.08	1 vs. 3	42.15	0.001^**^
			3	−0.36	0.07	2 vs. 3	6.70	0.010^*^
Equipment	22.47	0.001^**^	1	0.21	0.08	1 vs. 2	2.37	0.124
			2	0.02	0.07	1 vs. 3	21.89	0.001^**^
			3	−0.37	0.09	2 vs. 3	9.94	0.002^**^
Exhaustion	6.34	0.042^*^	1	−0.19	0.10	1 vs. 2	3.40	0.065
			2	0.08	0.10	1 vs. 3	5.62	0.018^*^
			3	0.14	0.11	2 vs. 3	0.18	0.674
Mental distance	4.44	0.109	1	−0.13	0.11	1 vs. 2	0.99	0.319
			2	0.03	0.11	1 vs. 3	4.44	0.035^*^
			3	0.18	0.11	2 vs. 3	0.86	0.353
Cognitive impairment	1.20	0.548	1	0.06	0.10	1 vs. 2	0.39	0.533
			2	−0.13	0.09	1 vs. 3	0.20	0.655
			3	0.13	0.10	2 vs. 3	1.20	0.274
Emotional impairment	0.81	0.666	1	0.02	0.09	1 vs. 2	0.01	0.905
			2	0.00	0.09	1 vs. 3	0.54	0.462
			3	0.11	0.09	2 vs. 3	0.62	0.431

^**^p < 0.01; ^*^p < 0.05; SE, standard error; Class 1, robust capability set; Class 2, inadequate capability set; Class 3, weak capability set.

As shown in Table 5, the robust class differed statistically significantly (*p* < 0.01) from the inadequate class regarding challenge demands, supervisor relations, autonomy, and equipment. The robust class also differed statistically significantly from the weak class concerning all four measured job resources, exhaustion, and mental distance. The inadequate and weak capability sets differed statistically significantly regarding challenge demands, autonomy, and equipment.

**Figure 3:**
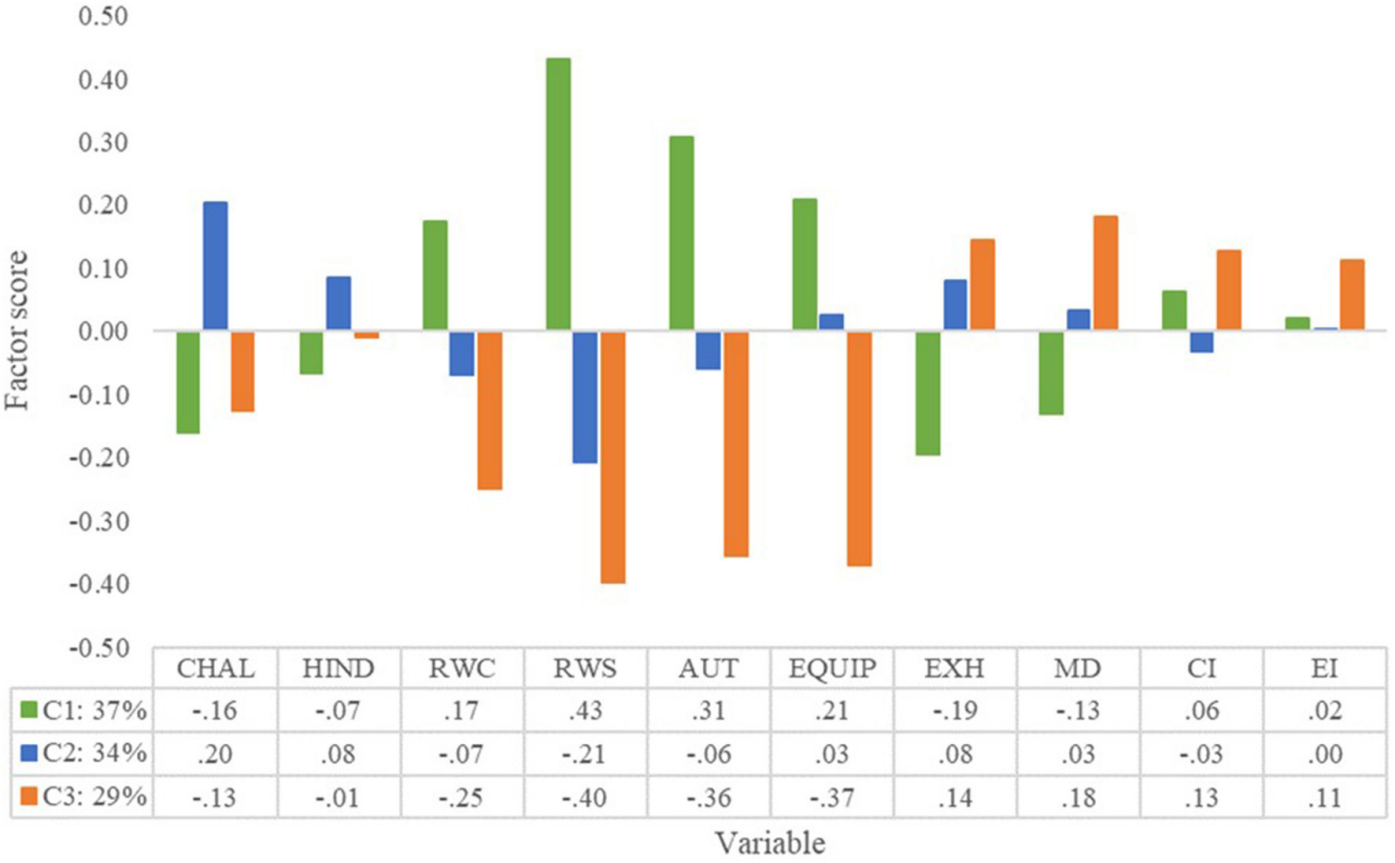
Capability classes, job demands and resources, and burnout of emergency nurses. CHAL, challenge demands; HIND, hindrance demands; RWC, relationship with co-workers; RWS, relationship with supervisor; AUT, autonomy at work; EQUIP, equipment; EXH, exhaustion; MD, mental distance; CI, cognitive impairment; EI, emotional impairment; C1, robust capability set; C2, inadequate capability set; C3, weak capability set.

Figure 3 shows that emergency nurses with inadequate capabilities experienced higher challenge demands than the other two classes. Emergency nurses with different capability sets differed most regarding their relationships with supervisors and co-workers, their autonomy at work, and equipment. Emergency nurses with the robust capability set reported better relationships with their supervisors and co-workers and higher job autonomy compared to the inadequate and weak capability sets. Furthermore, emergency nurses within the inadequate and weak capability sets (compared to the robust capability set) reported less equipment sufficiency. Hypothesis 2 is accepted. Regarding the four burnout components, the emergency nurse classes differed most on exhaustion and mental distance. The robust class had moderately low exhaustion and mental distance compared to the moderately high levels of the other two. Hypothesis 3 is, therefore, also partially accepted.

Multinomial regression analysis was used to study the differences among emergency nurses' capability sets and biographical information (see Table 6).

**Table 6:** Multinomial regression analysis of capability classes as dependent variable and biographical information as independent variable.

**Class**	**Variable**	**Gender**		**Education**		**Contract type**	
		**Estimate**	**SE**	**Estimate**	**SE**	**Estimate**	**SE**
Robust	Challenge	−0.04	0.08	0.02	0.02	−0.04	−0.04
	Hindrance	−0.14	0.10	0.03	0.03	0.01	−0.14
	Co-worker relations	0.05	0.10	−0.03	0.03	−0.02	0.05
	Supervisor relations	0.12	0.12	−0.06	0.03	0.02	0.12
	Autonomy	0.08	0.09	−0.03	0.03	0.03	0.08
	Equipment	−0.01	0.09	−0.06^*^	0.03	0.05	−0.01
	Exhaustion	−0.07	0.12	0.05	0.03	−0.09	−0.07
	Mental distance	−0.14	0.12	0.08	0.04	−0.09	−0.14
	Cognitive impairment	−0.17	0.11	0.09^*^	0.04	−0.03	−0.17
	Emotional impairment	−0.15	0.09	0.11^**^	0.03	−0.02	−0.15
Inadequate	Challenge	−0.04	0.08	0.02	0.02	−0.04	0.03
	Hindrance	−0.14	0.10	0.03	0.03	0.01	0.04
	Co-worker relations	0.05	0.10	−0.03	0.03	−0.02	0.05
	Supervisor relations	0.12	0.12	−0.06	0.03	0.02	0.05
	Autonomy	0.08	0.09	−0.03	0.03	0.03	0.04
	Equipment	−0.01	0.09	−0.06	0.03	0.05	0.04
	Exhaustion	−0.07	0.12	0.05	0.03	−0.09	0.05
	Mental distance	−0.14	0.12	0.08^*^	0.04	−0.09	0.05
	Cognitive impairment	−0.17	0.11	0.09^*^	0.04	−0.03	0.05
	Emotional impairment	−0.15	0.09	0.11^**^	0.03	−0.02	0.04
Weak	Challenge	−0.04	0.08	0.02	0.02	−0.04	0.03
	Hindrance	−0.14	0.10	0.03	0.03	0.01	0.04
	Co-worker relations	0.05	0.10	−0.03	0.03	−0.02	0.05
	Supervisor relations	0.12	0.12	−0.06	0.03	0.02	0.05
	Autonomy	0.08	0.09	−0.03	0.03	0.03	0.04
	Equipment	−0.01	0.09	−0.06^*^	0.03	0.05	0.04
	Exhaustion	−0.07	0.12	0.05	0.03	−0.09	0.05
	Mental distance	−0.14	0.12	0.08	0.04	−0.09	0.05
	Cognitive impairment	−0.17	0.11	0.09^*^	0.04	−0.03	0.05
	Emotional impairment	−0.15	0.09	0.11^**^	0.03	−0.02	0.04

^**^p < 0.01; ^*^p < 0.05.

From Table 6, it is evident that the measured emergency nurse biographical aspects did not differ statistically significantly (*p* < 0.01) between the job demands-resources and their capability set, nor between their capability set and the four burnout components, apart from their level of education (*r* = 0.11).

As evidenced in the findings, emergency nurses' challenge and hindrance demands are not significantly associated with the enablement and achievement of their work values, nor with them considering a work value as important. Regarding specific work capabilities, their challenge and hindrance demands were only significantly associated with building and maintaining meaningful relationships at work. Also, regarding the identified emergency nurse capability sets, those with inadequate capabilities experienced higher challenge demands than the other two classes. Therefore, the first hypothesis (H1) that emergency nurses' job demands are negatively associated with their work capabilities, is partially accepted.

Emergency nurses' job resources were significantly positively associated (*p* < 0.01) with the importance, enablement, and achievement work capability factors. Furthermore, job resources were positively negatively associated (*p* < 0.05) with all work capabilities, apart from relationship with co-workers and setting own goals. Furthermore, emergency nurses' capability sets differed most regarding their relationships with supervisors and co-workers, their autonomy at work, and equipment. Subsequently, Hypothesis 2 that emergency nurses' job resources are positively associated with their work capabilities, is accepted.

Regarding emergency nurses' burnout components, only exhaustion and mental distance where significantly negatively (*p* < 0.05) associated with the enablement and achievement components. There was only a significant negative relationship (*p* < 0.01) between exhaustion and mental distance, on the one hand, and earning a good income, on the other hand. Also, regarding the four burnout components, emergency nurses' work capability sets differed most on exhaustion and mental distance. Thus, the third hypothesis (H3) that emergency nurses' work capabilities are negatively related with their burnout, is partially accepted.

## 6. Discussion

This study investigated the sustainable employability of emergency nurses from the perspective of job demands and resources, work capabilities, and burnout. Emergency nurses' strongest capabilities were using knowledge and skills, developing knowledge and skills, and building and maintaining meaningful relationships at work. The weakest capabilities were earning a good income, involvement in decision making, and contributing to something valuable. Three distinct capability sets (namely, the robust, inadequate, and weak sets) were identified. Emergency nurses with a robust capability set (compared to those with inadequate and weak capability sets) reported significantly more job autonomy and better supervisor relations. Nurses with a weak capability set (compared to a robust capability set) reported significantly worse co-worker and supervisor relations, autonomy at work, and equipment sufficiency. Only the inadequate class exhibited challenge demands, with the robust class experiencing the lowest levels of exhaustion and mental distance.

All seven work capabilities were considered important by the emergency nurses. The most important value was using knowledge and skills, developing new knowledge and skills, and building and maintaining meaningful work relationships. Being involved in important decisions and contributing to something valuable were slightly less valued. Concerning enablement, using knowledge and skills, developing new knowledge and skills, and meaningful work relationships had the highest percentages, while earning a good income, involvement in decision making, and contributing to something valuable had the lowest percentages. The percentages of achievement showed the same pattern as enablement.

Emergency nurses' most robust capabilities were using knowledge and skills, developing new knowledge and skills, and building and maintaining meaningful relationships at work. However, substantial percentages of emergency nurses (32.35, 37.25, and 39.71%, respectively) lacked these capabilities. The weakest capabilities of emergency nurses included earning a good income, involvement in decision making, and contributing to something valuable. Large percentages of emergency nurses (76.96, 63.24, and 54.90%, respectively) lacked these capabilities. The higher prevalence of importance compared to enablement and achievement found among emergency nurses is in line with other populations and studies (Abma et al., 2016; Van Gorp et al., 2018; De Wet and Rothmann, 2022). Regarding specific capabilities, the results imply that three capabilities, namely, earning a good income, involvement in decision making, and contributing to something valuable, threaten the sustainable employability of emergency nurses. Given that more than 30% of the emergency nurses in this study lacked the four remaining capabilities presents a concern for their functioning and sustainable employability (van der Klink et al., 2016).

Emergency nurses' capability to build and maintain meaningful work relationships was negatively associated with challenge and hindrance demands. Theoretically, it makes sense that job demands results in fewer opportunities to build and maintain meaningful relationships, especially if emergency nurses value such relationships (Sen, 2009). Job autonomy (as a resource) seems to be essential for having capabilities such as using knowledge and skills, developing new knowledge and skills, being involved in important decisions, building and maintaining meaningful relationships at work, and contributing to something valuable. Job autonomy is regarded as a super capability that might encourage emergency nurses to convert demands and challenges into values (Robeyns, 2017; van der Klink, 2019). In line with the literature on social-structural and psychological empowerment (see Gloss et al., 2017), job autonomy also contributes to capabilities because it relates to emergency nurses' enablement and achievement. The CA has been related to the self-determination theory (Ryan et al., 2021), which highlights the importance of autonomy for human wellbeing. Moreover, Sen (1999) regarded freedom to do and be as a critical element in the CA. However, the results showed that emergency nurses' supervisor relationships are also associated with capabilities such as involvement in important decisions, building and maintaining meaningful relationships at work, earning a good income, and contributing to something valuable (Murangi et al., 2022).

Latent class analysis resulted in three capability sets for emergency nurses, namely, the robust, inadequate, and weak capability sets. Emergency nurses in the robust capability set scored high on six of the seven capabilities and obtained a moderate score for one capability (earning a good income). In contrast, nurses in the inadequate capability set showed high scores on using knowledge and skills, as well as developing new knowledge and skills. However, their capabilities to build and maintain meaningful work relationships, set own goals, involvement in important decisions, and earn a good income were relatively low. The weak capability set measured relatively low on all capabilities.

Concerning the job resources of the different capability sets, the most consistent differences between the three sets were found for two job resources, namely, supervisor relations and autonomy. Compared to the inadequate and weak capability sets, emergency nurses with a robust capability set had substantially more autonomy and better relationships with their supervisors. These two job resources were critical in converting emergency nurses' values into opportunities and, consequently, better functioning (van der Klink, 2019). Previous research indeed confirmed that the availability of job resources contributed to realizing work capabilities (Murangi et al., 2022). Therefore, in line with the CA (Sen, 1999), resources are associated with capabilities, i.e., opportunities to achieve actual freedom. However, resources are a means to an end, rather than opportunities (Sen, 1999; van der Klink, 2019).

Two job resources, namely, co-worker relations and equipment, were significantly different between the robust and the weak capability set classes. Emergency nurses in the weak capability set experienced poorer co-worker relations and indicated difficulties with the availability and quality of equipment they used in their jobs. Notably, emergency nurses in the weak class also lacked all seven capabilities, which made it difficult to convert resources into capabilities (Sen, 2009). Hindering job demands did not differ significantly between emergency nurses with different capability sets. However, emergency nurses in the inadequate capability set experienced higher challenge demands than those in the robust class. It is possible that the lower autonomy of the inadequate class and their poorer supervisor relations (which are critical to converting job demands into opportunities), combined with their capabilities to use knowledge and skills and develop new knowledge and skills, might result in more challenging demands (van der Klink, 2019).

This study confirmed the significant association of job demands and resources on burnout, in line with the findings of McDermid et al. (2020) and Lluch et al. (2022). The analyses showed that the capability set of emergency nurses and, notably, the lack of capabilities of the weak set (compared to the robust capability set) were associated with their exhaustion and mental distance. Considering the capabilities of emergency nurses in terms of what they value, what they are enabled for, and what they achieve is vital to protect them from burnout and ensure their sustainable employability.

The findings of this study contribute to the literature in the following ways: First, it provides evidence for a set of fundamental capabilities that is specific to the work of emergency nurses, which is essential for the theoretical development of the CA (see Gloss et al., 2017). The results showed the strongest and weakest capabilities of emergency nurses and the importance of a robust capability set. While capabilities regarding the use and development of knowledge and skills and meaningful work relationships will benefit the sustainable employability of emergency nurses, weaker capabilities in the set (e.g., earning a good income, involvement in decision making and contributing to something valuable) put emergency nurses' wellbeing at risk (Wolff and De-Shalit, 2007). Therefore, it is crucial for policymakers to equalize the capabilities (and not only resources) of everyone to enjoy valuable activities and states of being (Alkire et al., 2015). Second, the study resulted in new information regarding the associations between different capability sets and burnout of emergency nurses. Being able to do and to be (evident in the robust vs. the weak capability set) is essential to prevent burnout. Third, the study contributes new information about the relationship between specific job demands and resources and capability sets of emergency nurses. While resources are vital to counter emergency nurses' burnout, many are instrumental to individuals' other objectives but are not intrinsically valuable (Alkire et al., 2015). Also, individuals' abilities to convert resources into valued functionings vary. Job resources such as supervisor relations, autonomy, and co-worker relations do not only affect capabilities but might play an essential role in converting values into capabilities.

## 7. Practical implications

From the results, it was found that the measured job resources were significantly associated with the enablement and achievement work capability components and not the measured job demands. Consequently, it would probably be more beneficial to develop interventions aimed at improving the job resources available to emergency nurses in expanding their capability set (especially autonomy at work and relationship with supervisors) than in decreasing job demands (Schaufeli, 2017). Regarding emergency nurses' work capabilities, intervention should address their income and involvement in important decisions, as these were the two least prevalent capabilities among these professionals and the lowest within the three identified capability set classes. Given the significant and negative relationship between the measured job resources and emergency nurses' exhaustion and mental distance burnout components, it is recommended that intervention be aimed at improving emergency nurses' relationships with co-workers and supervisors, autonomy at work, and equipment sufficiency to lower their burnout levels. Furthermore, interventions aimed at addressing possible negative perceptions held by emergency nurses (e.g., whether they are indeed offered the opportunity to realize valued work functionings) could potentially lower their burnout levels. In this regard, a study among nursing students in the North of Spain found that higher perceptions of economic crisis were associated with higher burnout levels (Manzano-García et al., 2017). Therefore, emergency nurses with misperceptions about their work environment or available opportunities might benefit from interventions aimed at providing clarity on available resources and enabling factors offered by the hospital.

## 8. Limitations and recommendations for future research

This study had various limitations. Firstly, the cross-sectional design did not allow conclusions on the stability of the latent emergency nurses' capability sets over time. Longitudinal research would be beneficial (Spector, 2019). Secondly, although the significance of the study is in the novel context in which it investigated classes, the sample was drawn from a single province (i.e., Gauteng), mainly in the private sector. Future research including different samples (e.g., other provinces and the public sector) could strengthen the generalizability of the results. Thirdly, the sample size was a limitation. A larger group could potentially have produced additional latent emergency nurse capability set(s). In the fourth place, while the work values included in this study were considered sufficient (Abma et al., 2016; Murangi et al., 2022), unique work values might exist in a South African context. Therefore, qualitative studies of the work values of emergency nurses are essential.

## 9. Conclusion

Emergency nurses' predominant work capabilities were the use and development of knowledge and skills and having meaningful relationships at work, with earning a good income and being involved in important decisions being the lowest. Furthermore, three emergency nurse capability sets were found: a robust, an inadequate, and a weak capability set. The classes differed considerably on their relationships with co-workers and supervisors, autonomy at work, and equipment sufficiency, with only the inadequate class reporting challenge demands. Emergency nurses with a robust capability set reported lower exhaustion and mental distance than the other classes. Autonomy at work and relationship with supervisors were associated with the most work capabilities, with job demands only significantly associated with meaningful relationships at work. Earning a good income was significantly related to exhaustion and mental distance.

Emergency nurses considered most of the work values as being important. However, only a few were offered the opportunity in their work context to earn a good income. Fewer than half of the nurses were involved in important decisions, with a limited number of emergency nurses indicating they were earning a good income, were involved in important decisions, and were contributing to something valuable. Improving job resources (especially relationships with co-workers and supervisors, job autonomy, and equipment sufficiency) would increase emergency nurses' capabilities, decreasing their burnout levels, especially exhaustion and mental distance.

## Data availability statement

The original contributions presented in the study are publicly available. This data can be found here: https://data.mendeley.com/datasets/sngb8z87tw/1.

## Ethics statement

The studies involving human participants were reviewed and approved by North-West University Health Research Ethics Committee (NWU-HREC). The participants provided their written informed consent to participate in this study.

## Author contributions

NB collected and analyzed the data and wrote the paper. SR assisted in the data analyses and conceptualization, acted as an additional writer, and reviewed the paper. LD reviewed the data analyses, acted as an additional writer, and reviewed the paper. WL acted as an additional writer and reviewed the paper. All authors contributed to the article and approved the submitted version.

## Footnotes

^1^“Emergency nurse” refers to any nurse (professional or lower categories) working in the emergency department of a hospital providing Level 1 or Level 2 trauma care.
